# Rapid predictors for the occurrence of reduced left ventricular ejection fraction between LAD and non-LAD related ST-elevation myocardial infarction

**DOI:** 10.1186/s12872-015-0178-y

**Published:** 2016-01-05

**Authors:** Zhang-Wei Chen, Zi-Qing Yu, Hong-Bo Yang, Ying-Hua Chen, Ju-Ying Qian, Xian-Hong Shu, Jun-Bo Ge

**Affiliations:** Department of Cardiology, Shanghai Institute of Cardiovascular Diseases, Zhongshan Hospital, Fudan University, 180 Fenglin Road, Shanghai, 200032 PR China; Department of Endocrinology Medicine, East Hospital, Tongji University School of Medicine, Shanghai, 200120 China

**Keywords:** ST-elevation myocardial infarction, Left ventricular ejection fraction, Primary percutaneous coronary intervention, Predictors

## Abstract

**Backgrounds:**

Reduced left ventricular ejection fraction (LVEF) after acute myocardial infarction (AMI), which implies the occurrence of cardiac dysfunction, impacts cardiac prognosis, even after primary percutaneous coronary intervention (PCI). This study was designed to clarify the difference of clinical and angiographic predictors for reduced LVEF in ST-elevation myocardial infarction (STEMI) patients with left anterior descending artery (LAD) or non-LAD vessel as culprit artery.

**Methods:**

This was a retrospective study to review a total of 553 patients of STEMI underwent primary PCI in our hospital. All patients underwent echocardiography. Univariate analysis, multivariate analysis and classification and regression tree (CART) were performed between LAD related AMI and non-LAD related STEMI. The primary outcome was the occurrence of reduced LVEF 4–6 days after PCI.

**Results:**

In this study, culprit arteries of STEMI were 315 in LAD system (6 in left main artery, 309 in LAD) and 238 in non-LAD system (63 in left circumflex and 175 in right coronary artery). Compared with non-LAD group, post-MI LVEF was significantly reduced in LAD related STEMI group (52.4 ± 9.3 % vs. 57.1 ± 7.8 %, *P* < 0.01). Multivariate analysis indicated that elder (>65 years), time to hospital and proximal occlusion were associated with reduced LVEF (<55 %) in LAD related STEMI patients. However, in non-LAD patients, time to hospital, multivessel stenosis and post-PCI blood pressure predicted the occurrence of reduced LVEF. Furthermore, CART analysis also obtained similar findings.

**Conclusions:**

Patients with LAD or non-LAD related STEMI could suffer reduced LVEF, while the clinical and angiographic predictors for the occurrence were different.

## Background

Reduced left ventricular ejection fraction (LVEF), which is significantly associated with cardiac dysfunction, occurs approximately in 30–40 % of patients who suffer ST-elevation myocardial infarction (STEMI). Although the incidence of reduced LVEF after STEMI declined significantly [1] because of great advancement in the treatment of anti-thrombotic therapy and primary percutaneous coronary intervention (PCI), it is still one of most critical complications after STEMI that carries a poor cardiac prognosis [2–7]. Reduced LVEF, which is common in STEMI patients with left main artery (LM) or left anterior descending artery (LAD) as culprit vessel. The occurrence was related to older ages, hypertension, diabetes [1], time to reperfusion [8], and higher prevalence of proximal occlusion [9]. The extent of acute myocardial damage in LAD occlusion is markedly larger than that in either left circumflex (LCX) or right coronary artery (RCA) occlusion. However, it has been also reported that reduced LVEF or cardiac dysfunction occurred in STEMI patients with RCA or LCX as culprit vessel [10, 11]. However, it was unclear whether there were predictors difference for reduced LVEF between LAD-related and non-LAD-related STEMI.

Given to the impact of new-onset cardiac dysfunction after STEMI on the cardiac prognosis, it is important to investigate the difference of clinical and angiographic predictors for reduced LVEF in STEMI patients with different culprit vessels. These findings will provide rapid prediction and beneficial effects on the early prevention of cardiac dysfunction after STEMI.

## Methods

### Study population

This was a retrospective clinical study to review the patients of acute STEMI underwent primary PCI (*n* = 664) in our hospital from Jul 2011 to Oct 2013. Baseline 12-lead electrocardiograms were performed at admission. A total of 553 patients were included in this study, while 111 patients were excluded according to follow exclusion criteria. The inclusion criteria were: (1) patients with 18 to 85 years of age; (2) diagnosed as acute STEMI; (3) underwent primary coronary intervention within 12 h after chest pain on-set. The exclusion criteria were as follows: (1) incomplete clinical history record (*n* = 23); (2) excluded the diagnosis of myocardial infarction by angiography, such as viral myocarditis, pericarditis or cardiomyopathy (*n* = 26); (3) not finish the detection of echocardiography in-hospitalization because of any reason (*n* = 17); (4) confirmed clinical heart failure before this admission, complicated with cardiomyopathy, congenital heart diseases and rheumatic heart disease (*n* = 19); (5) active chronic inflammation (*n* = 14); (6) dysfunction of hematological and immunological system (*n* = 4); (7) carcinoma or a condition treated with immunosuppressive agents (*n* = 8). This study and consent procedure were approved by our local ethics committee (Ethics Committee of Zhongshan Hospital affiliated to Fudan University), and were carried out in accordance with the principles of the Declaration of Helsinki. Consent for publication of these data was obtained from each patient when they were admitted in our hospital.

Several important clinical variables were record, such as age, gender, hypertension, diabetes, stable angina history, Time to hospital (from chest pain on-set to diagnosis) and D-to-B time (door to balloon).

### Primary percutaneous coronary intervention

Prior to primary PCI, all patients received adequate loading doses of aspirin (300 mg) and clopidogrel (300 mg) or ticagrelor (180 mg) immediately after diagnosed as STEMI. Procedure of PCI was performed immediately via the femoral or radial access route. The characteristics of coronary angiography were record, such as culprit artery, acute occlusive segment and multi-vessel disease (defined as having at least another vessel with 75 % or greater stenosis except the culprit occlusion artery, such as culprit vessel in LAD had LCX or RCA or LM stenosis, culprit vessel in LCX had RCA or LAD or LM stenosis, culprit vessel in RCA had LCX or LAD or LM stenosis). A lesion was considered proximal if it was located proximal to the first diagonal branch in the LAD, the first obtuse marginal branch in LCX, or the first acute marginal branch in RCA [12].

The usages of interventional techniques, thrombus aspiration and platelet glycoprotein IIb/IIIa receptor inhibitor were chosen at the operators’ discretion. The phenomenon of no reflow or slow flow post-stenting was record. Post-PCI blood pressure, including systolic blood pressure (SBP) and diastolic blood pressure (DBP), was detected by invasive blood pressure monitor from radial or femoral vascular sheaths.

### Echocardiography

Echocardiography was performed before discharge (4–6 days post-PCI) in all patients using a Philips IE33 instrument (Philips, Netherlands) with a 2–3.5 MHz transducer (X3-1), while left ventricular ejection fraction (LVEF) were detected by Simpson method. It was defined as reduced LVEF while LVEF less than 55 %. All exams were performed by one of three echocardiography operators. These three operators underwent standardized training before this study. Observers who detected LVEF were blinded to the results of coronary angiography and clinical record. The incidence of reduced LVEF after STEMI was compared between two different culprit artery systems, including LAD related STEMI (LM occlusion and LAD occlusion, *n* = 315) and non-LAD related STEMI (LCX occlusion, *n* = 63; RCA occlusion, *n* = 175).

### Statistical Analysis

All statistical analyses were performed with SPSS software 19.0. Data were presented as the percentage or mean ± standard deviation (SD). *Chi*-square analysis was used to compare the frequency for categorical variables, and Student’s *t* or correction *t* tests were used to compare means for continuous variables. Multivariable logistic analysis was performed to identify the independent risk factors for reduced LVEF (LVEF < 55 %). Stratification according to different risk subsets was also made by classification and regression tree (CART) analysis. All *P*-values were two-sided, and *P* < 0.05 was considered to indicate statistical significance.

## Results

### Clinical and angiographic characteristics

A total of 553 STEMI patients enrolled with average age 64.0 ± 12.0 years. There were 447 men (80.8 %) and 106 women (19.2 %). The prevalence of hypertension and diabetes were 60.4 % (334 patients) and 48.8 % (270 patients), respectively. Culprit arteries of STEMI were 6 in LM, 309 in LAD, 63 in LCX and 175 in RCA, respectively. Baseline clinical and angiographic characteristics of patients with different culprit arteries were shown in Table 1. Compared with the non-LAD related STEMI (culprit arteries were RCA and LCX), patients of LAD related STEMI (culprit arteries were LM and LAD) had lower LVEF (52.4 ± 9.3 % vs. 57.1 ± 7.8 %, *P* < 0.01) and higher incidence of reduced LVEF (LVEF < 55 %: 53.7 and 26.9 %, *P* < 0.01).

**Table 1:** Comparison of clinical and angiographic characteristics among STEMI patients with different culprit vessel

	LAD system (*n* = 315)			Non-LAD system (*n* = 238)		
	EF < 55 %	EF ≥ 55 %	P	EF < 55 %	EF ≥ 55 %	P
	*n* = 169	*n* = 146		*n* = 64	*n* = 174	
(1) Clinical characteristics						
Male (%)	133 (78.7 %)	121 (82.9 %)	0.349	54 (84.4 %)	139 (79.9 %)	0.433
Age (years)	65.4 ± 11.4	60.7 ± 12.7	<0.01	67.4 ± 11.9	63.9 ± 11.3	0.034
Hypertension (%)	98 (58.7 %)	82 (56.6 %)	0.704	40 (62.5 %)	114 (65.9 %)	0.627
Diabetes (%)	94 (55.6 %)	68 (46.6 %)	0.098	29 (45.3 %)	79 (45.7 %)	0.961
Stable angina history (%)	58 (34.3)	59 (40.4 %)	0.265	25 (39.1 %)	62 (35.6 %)	0.626
Time to hospital (hours)	6.0 ± 2.5	5.3 ± 2.7	<0.01	6.6 ± 2.7	5.3 ± 2.4	<0.01
D-to-B time (minutes)	76.2 ± 27.4	74.6 ± 25.6	0.675	71.2 ± 21.3	74.3 ± 22.7	0.304
(2) Angiographic characteristics						
Number of disease vessels	1.8 ± 0.8	1.6 ± 0.8	0.197	2.2 ± 0.8	1.9 ± 0.8	0.061
Multi-vessel stenosis (%)	94 (55.6 %)	66 (45.2 %)	0.065	47 (73.4 %)	100 (57.5 %)	0.025
--two vessels disease (including culprit vessel)	56	35		19	51	
--three vessels disease (including culprit vessel)	38	31		28	49	
--LAD AMI complicated with LCX stenosis	64	46		--	--	
--LAD AMI complicated with RCA stenosis	68	51		--	--	
--non-LAD AMI complicated with LAD stenosis	--	--		24	48	
Occlusion in proximal segment (%)	101 (59.8 %)	70 (47.9 %)	0.036	21 (32.8 %)	57 (32.8)	0.994
Slow or no reflow (%)	34 (20.1 %)	26 (17.8 %)	0.603	12 (18.8 %)	20 (11.5 %)	0.146
Post-PCI SBP (mmHg)	114.5 ± 16.8	119.2 ± 16.7	0.014	110.7 ± 18.7	113.1 ± 16.5	0.368
Post-PCI DBP (mmHg)	70.7 ± 9.1	72.9 ± 7.7	0.027	68.5 ± 11.0	70.9 ± 9.0	0.123

*LAD system* STEMI in left main or left main artery or left anterior descending artery; *Non-LAD system* STEMI in left circumflex or right coronary artery;

*DBP* diastolic blood pressure; *D-to-B* door to balloon; *SBP* systolic blood pressure;

*STEMI* ST-Elevation Myocardial Infarction; Time to hospital: from chest pain on-set to diagnosis;

### Reduced LVEF and predictor analysis

**Fig. 1:**
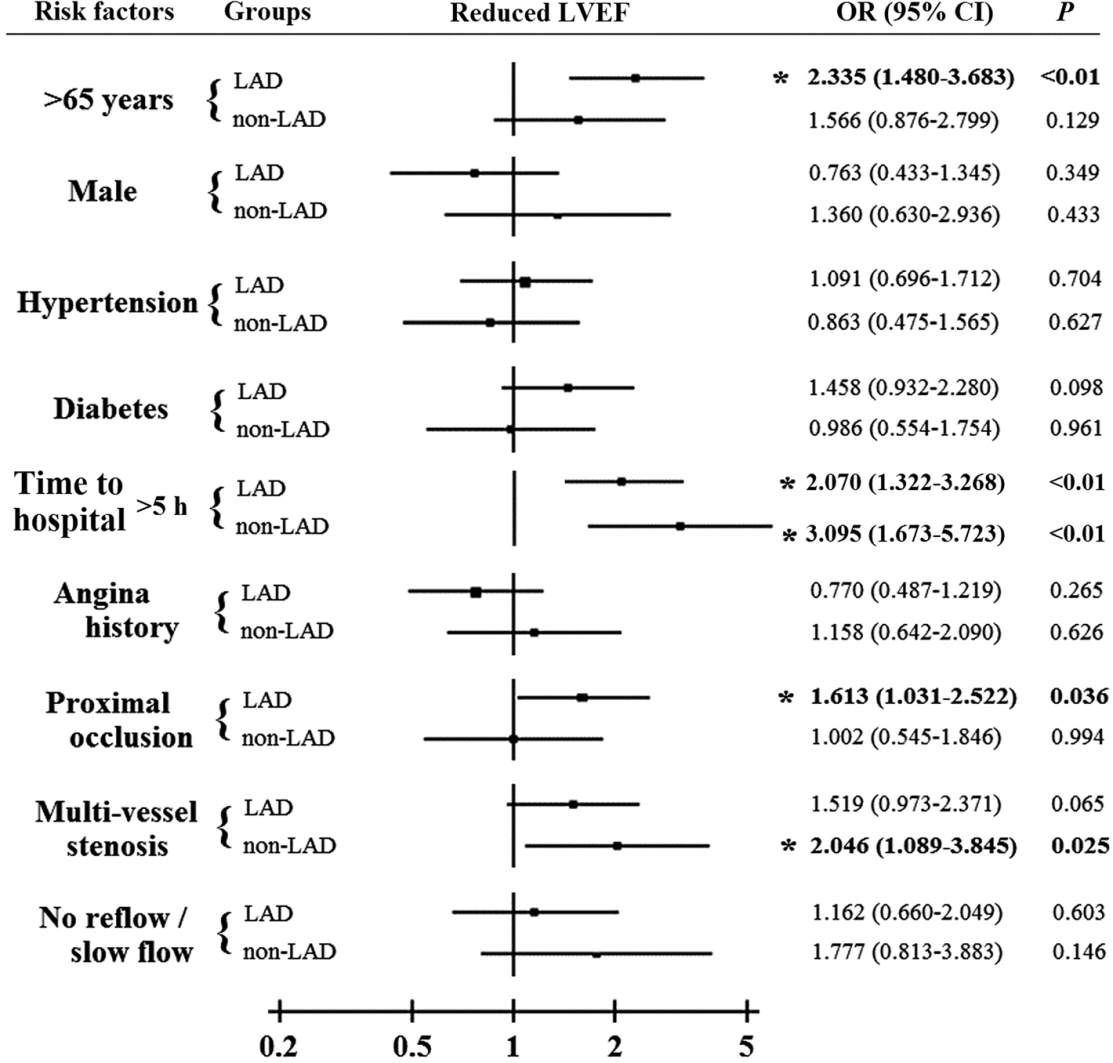
Subgroups analysis of clinical and angiographic factors for the increasing risk of reduced LVEF (LVEF < 55 %) in LAD and non-LAD related STEMI groups

**Fig. 2:**
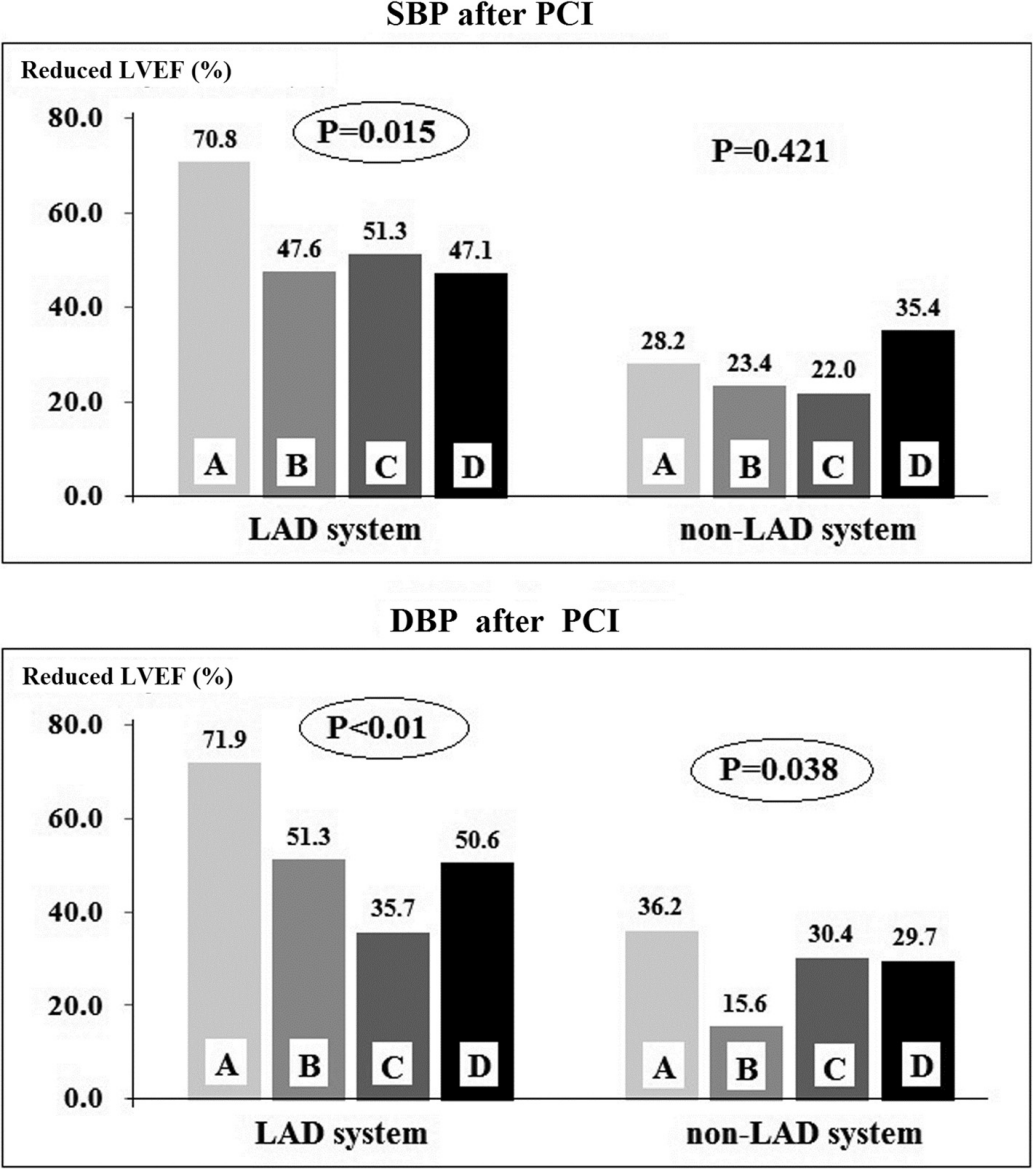
LAD system: STEMI in left main or left main artery or left anterior descending artery; Non-LAD system: STEMI in left circumflex or right coronary artery; DBP: diastolic blood pressure; SBP: systolic blood pressure; Groups A to D indicated four groups classified by the quartile of post-PCI blood pressure SBP: group A: <102mmHg; Group B: 103-110mmHg; Group C: 111-120mmHg; Group D: >120mmHg DBP: group A: <65mmHg; Group B: 66-70mmHg; Group C: 71-78mmHg; Group D: >79mmHg. The incidence of reduced LVEF in different post-PCI blood pressure subgroups

In order to clarify the predictor difference for reduced LVEF in LAD system and non-LAD system groups, several clinical and angiographic predictors were analyzed by univariate analysis, shown in Table 1 and Fig. 1. It was demonstrated that elder (more than 65 years), time to hospital (from chest pain on-set to diagnosis), acute occlusion in proximal segment and post-PCI blood pressure significantly increased the risk of reduced in LAD system. However, in non-LAD related STEMI patients, beside the factors of age and time to hospital, multivessel stenosis significantly increased the risk of reduced LVEF. In order to further clarify the influence of post-PCI blood pressure, the occurrence of reduced LVEF was analyzed among four groups classified by the quartile of post-PCI blood pressure in patients with LAD system or non-LAD system STEMI, shown in Fig. 2. We found that lower SBP and DBP after primary PCI predicted the higher risk of reduced LVEF.

### Multivariate analysis

Multivariate logistic analysis was performed to demonstrate the independent effect of these predictors (confirmed statistic difference in univariate analysis) on the occurrence of reduced LVEF after STEMI, In this analysis, reduced LVEF (LVEF < 55 %) was employed as a dependent variable in both LAD and non-LAD system subgroups, while age > 65 years, multi-vessel stenosis, acute occlusion in proximal segment, time to hospital, post-PCI SBP <100 mmHg and post DBP <65 mmHg were set as independent variables, shown in Table 2. These results demonstrated that elder (OR = 1.984, 95 % CI = 1.205–3.266, *P* < 0.01), proximal occlusion (OR = 1.681, 95 % CI = 1.042–2.713, *P* = 0.033) and time to hospital (OR = 1.106, 95 % CI = 1.010–1.210, *P* = 0.029) were major independent predictors for reduced LVEF in LAD system, while time to hospital (OR = 1.246, 95 % CI = 1.097–1.414, *P* < 0.01), multi-vessel stenosis (OR = 2.394, 95 % CI = 1.185–4.836, *P* = 0.015) and post-PCI SBP < 100 mmHg (OR = 2.927, 95 % CI = 1.052121–7.643, *P* = 0.028) in non-LAD system.

**Table 2:** Odds ratios of independent predictors for reduced LVEF after STEMI in LAD and non-LAD system (multivariate logistic analysis)

Predictors	OR	95 % confidence intervals	P
LAD system			
Elder (>65 years)	1.984	1.205–3.266	<0.01
Proximal occlusion	1.681	1.042–2.713	0.033
Time to hospital	1.106	1.010–1.210	0.029
Multi-vessel stenosis	1.395	0.848–2.296	0.190
Post-PCI SBP < 100 mmHg	1.563	0.540–4.521	0.410
Post-PCI DBP < 60 mmHg	1.677	0.778–3.613	0.187
Non-LAD system			
Elder (>65 years)	1.167	0.616–2.209	0.635
Proximal occlusion	1.108	0.569–2.159	0.762
Time to hospital	1.246	1.097–1.414	<0.01
Multi-vessel stenosis	2.394	1.185–4.836	0.015
Post-PCI SBP < 100 mmHg	2.927	1.121–7.643	0.028
Post-PCI DBP < 60 mmHg	1.778	0.792–3.988	0.163

*LAD system* STEMI in left main or left main artery or left anterior descending artery;

*Non-LAD system* STEMI in left circumflex or right coronary artery;

*DBP* diastolic blood pressure; *SBP* systolic blood pressure;

*STEMI* ST-Elevation Myocardial Infarction;

Time to hospital: from chest pain on-set to diagnosis

### CART analysis

**Fig. 3:**
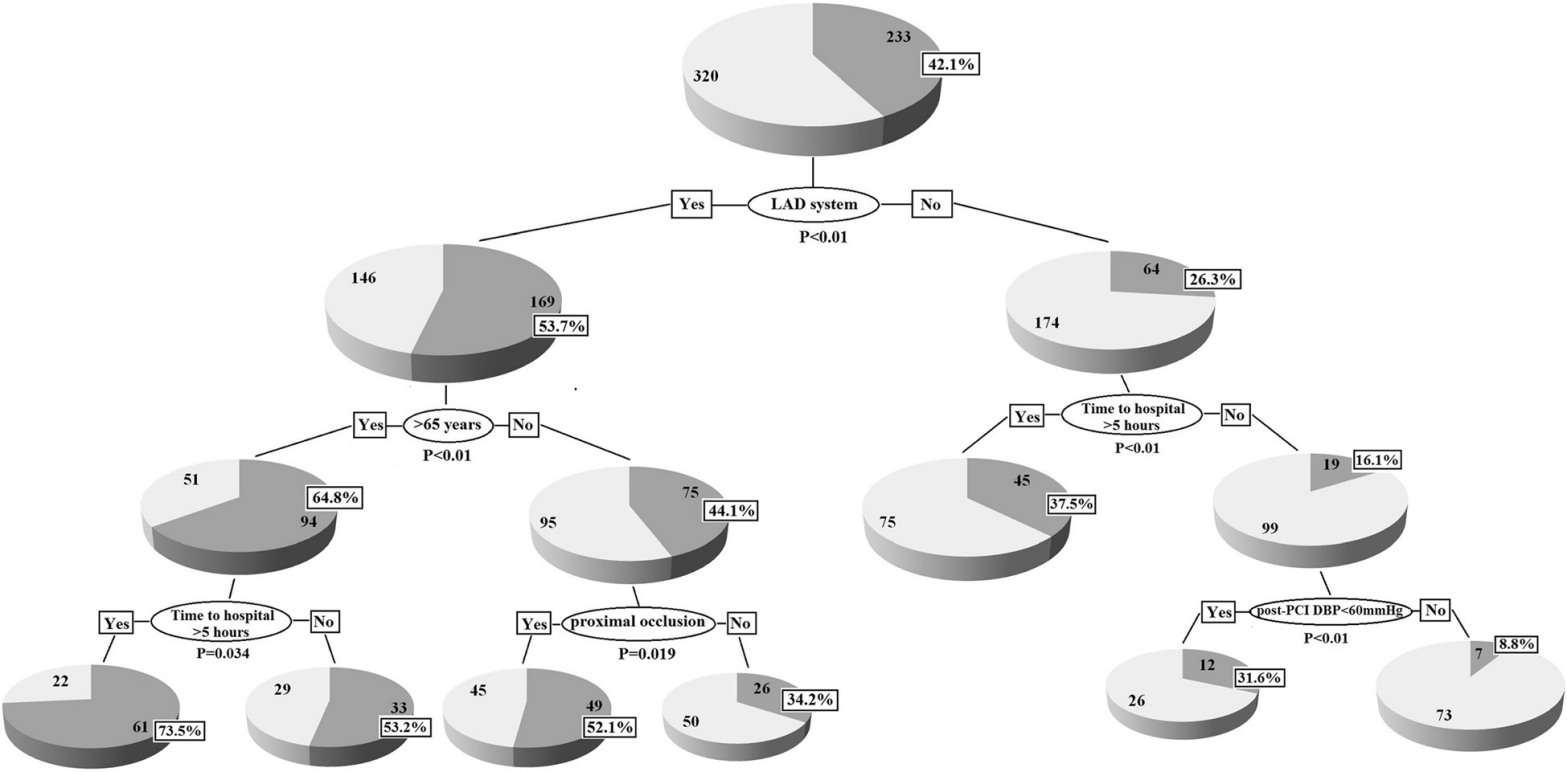
CART analysis demonstrated the major predictors and diagnostic steps for reduced LVEF

In order to confirm the impact of predictors on reduced LVEF and simply the prediction process, CART analysis was also applied to assess the incidence of reduced LVEF after STEMI in multivariate subgroups. Reduced LVEF was employed as a dependent variable, while age > 65 years, male gender, stable angina history, diabetes, hypertension, culprit vessel (LAD system or non-LAD system), time to hospital >5 h, multi-vessel stenosis, occlusion in proximal segment, slow or no reflow, post-PCI SBP <100 mmHg and post DBP < 65 mmHg were set as independent variables. CART analysis results were shown in Fig. 3. We found that LAD system was the major determinant of reduced LVEF after STEMI. Beside culprit artery, elder, time to hospital > 5 h and proximal occlusion were most critical three steps in risk stratification for reduced LVEF in LAD system, while time to hospital and post-PCI diastolic blood pressure < 60 mmHg in non-LAD system.

## Discussion

Acute myocardial infarction (AMI), which is mostly caused by coronary plaque rupture or erosion, could result in several clinical complications and impact cardiac prognosis [4, 13]. Reduce LVEF or cardiac dysfunction occurs approximately in 30–40 % of patients who suffer STEMI, and the mortality of patients with post-MI cardiac dysfunction is 20 to 30 % [7]. As we know, reduced LVEF was common in STEMI patients with LM or LAD as culprit vessel. It has also been reported that reduced LVEF could occurred in patients with RCA or LCX as culprit vessel [10, 11, 14]. However, there were few studies focused on the risk factors or clinical predictors for reduced LVEF caused by RCA or LCX-related MI. Furthermore, it was unclear whether there were different clinical and angiographic characteristics between LAD and non-LAD-related STEMI with reduced LVEF.

In this study, we confirmed the occurrence of reduced LVEF (LVEF < 55 %) in non-LAD related MI patients, although this prevalence was lower than that in LAD related MI group (26.9 vs. 53.7 %, *P* < 0.01). In order to clarify the difference of predictors between these two different culprit vessels, sub-group analyses and multivariate logistic analysis were also performed. We found that elder age, Time to hospital and proximal occlusion were critical for reduced LVEF in LAD related STEMI, while multi-vessel stenosis, Time to hospital and post-PCI blood pressure contributed most to reduce LVEF in non-LAD related STEMI.

De Luca G reported [15] that elderly patients complicated with higher incidence of hypertension and diabetes, more advanced Killip class at presentation, longer time to treatment, higher prevalence of distal embolization and significantly impaired myocardial perfusion, which resulted in worse coronary microcirculation and higher mortality after STEMI [16], even undergoing primary angioplasty. Previously, few studies reported the different impact of older age on cardiac dysfunction between LAD and non-LAD related STEMI. In the LAD related MI, significantly impaired microcirculation and proximal occlusion directly resulted in more severe ischemia and large area of infarction, which exacerbated left ventricular systolic dysfunction. However, on the contrary, the extent of myocardial perfusion injury in non-LAD vessels contributes less to the left ventricular systolic function. In the non-LAD related MI, multivessel stenosis, which contained LAD stenosis, was more important for left ventricular function.

Hypertension has also been one of well-established factors for increasing risk of cardiovascular diseases, such as acute myocardial infarction and heart failure [17]. Meanwhile, low admission blood pressure in MI patients has been suggested as a predictor for cardiac mortality [18]. However, it was unclear about the predictive value of post-PCI blood pressure on the risk of reduced LVEF between different culprit arteries. In our study, we found that lower post-PCI systolic (<100 mmHg) or diastolic (<60 mmHg) blood pressure indicated the higher incidence of reduced LVEF (Fig. 2). After adjusted by other factors, post-PCI systolic blood pressure was also independently associated with cardiac dysfunction in non-LAD related MI.

As we know, reperfusion time to STEMI is one of the most important factors for short- and long-term cardiac prognosis [19]. In our study, there was no significant difference of D-to-B time between cardiac dysfunction and preserved function groups. However, time from chest pain on-set to diagnosis, defined as Time to hospital, was quite different between these two groups. As demonstrated in multivariable analysis, STEMI patients with Time to hospital more than 5 h had less LVEF no matter in LAD or non-LAD related STEMI groups, which could be resulted in more injured cardiomyocytes and higher risk of cardiac complications. CART analysis, which was analyzed based different risk factors’ subgroups, indicated rapid prediction for the occurrence of reduced LVEF.

We should note some of our study’s limitations. First, the number of included patients was small size. Second, its retrospective nature limited its potency to clarify the cause relation between predictions and reduced LVEF. Third, the inclusion of LVEF in this study was only short-term data, therefore, long-term data will be needed to document the association between predictors and post-PCI LVEF. These limitations will be taken into account in our further clinical researches and prospective studies.

## Conclusions

Patients with LAD or non-LAD related STEMI could suffer reduced LVEF, while the clinical and angiographic predictors for the occurrence were quite different.

## Abbreviations

AMI: Acute myocardial infarction
CAD: Coronary artery disease
CART: Classification and regression tree
LAD: Left anterior descending artery
LCX: Left circumflex
LVEF: Left ventricular ejection fraction
PCI: Percutaneous coronary intervention
RCA: Right coronary artery
STEMI: ST-elevation myocardial infarction
